# Penta­ammonium hepta­sodium bis­[penta­kis(μ_2_-oxido)deca­oxido­bis­(μ_5_-phosphato)penta­molybdenum(VI)] henicosa­hydrate

**DOI:** 10.1107/S160053681000601X

**Published:** 2010-02-20

**Authors:** Hssain Bih, Lahcen Bih, Bouchaid Manoun, Mohamed Azrour, Peter Lazor, Lahcen El Ammari

**Affiliations:** a Laboratoire de Chimie des Matériaux et de l’Environnement, FSTG-Marrakech, Morocco; b Equipe Sciences des Matériaux, Faculté des Sciences et Techniques, Errachidia, Morocco; c Department of Earth-Geology, Uppsala University, Sweden; d Laboratoire de Chimie du Solide Appliquée, Faculté des Sciences, Université Mohammed V-Agdal, Avenue Ibn Battouta, BP 1014, Rabat, Morocco

## Abstract

The title compound, (NH_4_)_5_Na_7_[Mo_5_P_2_O_23_]_2_·21H_2_O, was prepared under atmospheric conditions in aqueous solution at room temperature. The structure contains the [Mo_5_P_2_O_23_]^6−^ heteropolyoxometallate anion, which has been previously reported a number of times with a variety of differing counter-cations. Each anion is built up of five MoO_6_ octa­hedra sharing an edge and forming a ring which is closed by common corners of the terminal octa­hedra. The rings are closed on both sides by two asymmetric PO_4_ tetra­hedra, sharing three corners with three MoO_6_ octa­hedra. The anions are chiral and the two independent anions in the asymmetric unit were arbitarily chosen with the same chirality, but the centrosymmetric crystal contains both enanti­omers. The structure can alternatively be described as a succession of layers parallel to (101), formed by the [Mo_5_P_2_O_23_]^6−^ anions and linked by sodium chains. Water mol­ecules and ammonium ions fill the remaining space and ensure the cohesion through extensive N—H⋯O and O—H⋯O hydrogen bonding.

## Related literature

For ammonium polyoxomolybophosphates, see: Boeyens *et al.* (1976 ▶); Ferrari & Nanni (1939 ▶); Ilhan *et al.* (2007 ▶); Andersen & Villadsen (1993 ▶); Xu *et al.*(1998 ▶). For background to the heteropolyoxometallate anion, see: Hedman & Strandberg (1979 ▶); Long *et al.* (2007 ▶); Pope (1983 ▶); Strandberg (1973 ▶). For examples of hybrid compounds see: Ma *et al.* (2006 ▶); Wu *et al.* (2009 ▶).
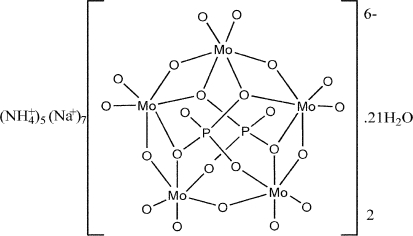


## Experimental

### 

#### Crystal data

(NH_4_)_5_Na_7_[Mo_5_P_2_O_23_]_2_·21H_2_O
*M* *_r_* = 2448.76
Triclinic, 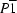

*a* = 9.2299 (3) Å
*b* = 18.3516 (6) Å
*c* = 19.7918 (6) Å
α = 73.860 (1)°
β = 85.323 (3)°
γ = 75.772 (2)°
*V* = 3121.17 (17) Å^3^
*Z* = 2
Mo *K*α radiation
μ = 2.23 mm^−1^
*T* = 298 K
0.42 × 0.14 × 0.08 mm

#### Data collection

Bruker X8 APEXII Diffractometer
Absorption correction: multi-scan (*SADABS*; Bruker, 2005 ▶) *T*_min_ = 0.695, *T*_max_ = 0.837
132450 measured reflections
33626 independent reflections
28095 reflections with *I* > 2σ(*I*)
*R*_int_ = 0.024

#### Refinement

*R*[*F* ^2^ > 2σ(*F* ^2^)] = 0.022
*wR*(*F* ^2^) = 0.057
*S* = 1.09
33626 reflections
839 parameters
H-atom parameters constrained
Δρ_max_ = 0.99 e Å^−3^
Δρ_min_ = −0.76 e Å^−3^

### 

Data collection: *APEX2* (Bruker, 2005 ▶); cell refinement: *SAINT* (Bruker, 2005 ▶); data reduction: *SAINT*; program(s) used to solve structure: *SHELXS97* (Sheldrick, 2008 ▶); program(s) used to refine structure: *SHELXL97* (Sheldrick, 2008 ▶); molecular graphics: *ORTEP-3 for Windows* (Farrugia, 1997 ▶) and *PLATON* (Spek, 2009 ▶); software used to prepare material for publication: *WinGX* publication routines (Farrugia, 1999 ▶).

## Supplementary Material

Crystal structure: contains datablocks I, New_Global_Publ_Block. DOI: 10.1107/S160053681000601X/fj2276sup1.cif

Structure factors: contains datablocks I. DOI: 10.1107/S160053681000601X/fj2276Isup2.hkl

Additional supplementary materials:  crystallographic information; 3D view; checkCIF report

## Figures and Tables

**Table 1:** Hydrogen-bond geometry (Å, °)

*D*—H⋯*A*	*D*—H	H⋯*A*	*D*⋯*A*	*D*—H⋯*A*
O1—H11⋯O12*B*^i^	0.86	2.02	2.8415 (18)	160
N1—H11*N*⋯O12*A*	0.87	2.07	2.750 (2)	135
O1—H12⋯O16*A*^ii^	0.86	2.55	3.383 (2)	164
N1—H12*N*⋯O20	0.87	2.13	2.963 (3)	161
N1—H13*N*⋯O18*B*^iii^	0.87	2.37	2.909 (2)	121
N1—H14*N*⋯O23*B*^iii^	0.87	2.29	2.922 (2)	130
O2—H21⋯O21*B*^iv^	0.86	2.29	3.1414 (17)	172
N2—H21*N*⋯O5*A*^ii^	0.87	2.10	2.8892 (19)	151
N2—H21*N*⋯O13*A*	0.87	2.59	3.146 (2)	123
O2—H22⋯O20*A*	0.86	2.07	2.9134 (18)	165
N2—H22*N*⋯O9*A*	0.87	2.20	3.036 (2)	162
N2—H23*N*⋯O1*A*	0.87	1.98	2.837 (2)	171
N2—H24*N*⋯O15*A*^ii^	0.87	2.06	2.9121 (19)	167
O3—H31⋯O23*A*^iii^	0.86	1.86	2.7120 (16)	174
N3—H31*N*⋯O5*B*^v^	0.87	1.95	2.8066 (19)	170
O3—H32⋯O14*B*^iii^	0.86	2.00	2.8440 (16)	165
N3—H32*N*⋯O9*B*^iii^	0.87	2.09	2.9533 (19)	173
N3—H33*N*⋯O1*B*^iii^	0.87	2.04	2.8410 (19)	152
N3—H34*N*⋯O15*B*^v^	0.87	2.19	3.017 (2)	159
O4—H41⋯O10^ii^	0.86	1.89	2.743 (2)	172
N4—H41*N*⋯O1*A*	0.87	2.00	2.8544 (19)	171
O4—H42⋯O23*A*	0.86	2.57	3.403 (2)	164
N4—H42*N*⋯O5*A*^ii^	0.87	2.02	2.8757 (19)	169
N4—H43*N*⋯O12*A*^iii^	0.87	2.44	3.123 (2)	136
N4—H43*N*⋯O22*A*	0.87	2.26	2.9312 (19)	134
N4—H44*N*⋯O17*A*^iii^	0.87	2.23	2.958 (2)	142
O5—H51⋯O9*A*	0.86	2.40	3.0367 (18)	132
O5—H51⋯O15*B*^vi^	0.86	2.42	3.201 (2)	151
N5—H51*N*⋯O1*B*^iii^	0.87	2.03	2.8649 (19)	162
O5—H52⋯O2*A*	0.86	1.97	2.8118 (18)	167
N5—H52*N*⋯O5*B*^v^	0.87	1.96	2.8251 (17)	175
N5—H53*N*⋯O13*B*^iii^	0.87	2.55	2.9140 (18)	106
N5—H54*N*⋯O21*B*^v^	0.87	2.45	3.0098 (18)	123
N5—H54*N*⋯O17*B*^i^	0.87	2.28	3.0203 (19)	144
O6—H61⋯O10*A*	0.86	2.01	2.8234 (17)	159
O6—H62⋯O20*B*^iv^	0.86	1.84	2.6971 (16)	176
O7—H71⋯O3^iii^	0.86	1.89	2.7347 (19)	167
O7—H72⋯O6*A*^vii^	0.86	2.09	2.9430 (16)	173
O8—H81⋯O6	0.86	1.87	2.7221 (19)	174
O8—H82⋯O2*B*^vii^	0.86	2.01	2.8642 (16)	171
O9—H91⋯O5*A*^vii^	0.86	1.90	2.7476 (18)	171
O9—H92⋯O13*A*^vi^	0.86	2.34	2.9118 (19)	124
O9—H92⋯O14*B*^vi^	0.86	2.58	3.0630 (18)	117
O1—H01⋯O9*B*^vii^	0.86	2.20	2.985 (2)	152
O10—H102⋯O6*A*	0.86	2.10	2.893 (2)	153
O11—H111⋯O17*A*^viii^	0.86	2.58	2.987 (2)	110
O11—H112⋯O17*A*^viii^	0.86	2.58	2.987 (2)	110
O11—H112⋯O21*A*	0.86	2.39	3.126 (2)	143
O12—H121⋯O9^ix^	0.86	2.04	2.891 (2)	171
O12—H122⋯O18*A*^iii^	0.86	2.01	2.834 (2)	160
O13—H131⋯O19*A*^ix^	0.86	2.13	2.944 (2)	158
O13—H132⋯O2*B*^iii^	0.86	2.12	2.904 (2)	151
O14—H141⋯O11	0.86	1.92	2.772 (2)	171
O14—H142⋯O6*B*^iii^	0.86	2.17	2.9073 (18)	143
O15—H151⋯O6*B*^vi^	0.86	2.01	2.8578 (17)	169
O15—H152⋯O15*B*^vi^	0.86	2.37	3.0264 (18)	134
O15—H152⋯O9*A*	0.86	2.43	3.192 (2)	148
O16—H161⋯O13^x^	0.86	1.92	2.769 (2)	171
O16—H162⋯O20*B*^iv^	0.86	2.39	3.220 (2)	163
O17—H171⋯O1*B*^i^	0.86	2.44	3.167 (2)	143
O17—H172⋯O16*B*^iv^	0.86	2.18	2.949 (2)	149
O18—H181⋯O1*A*	0.86	2.35	3.105 (2)	147
O18—H182⋯O11*B*^i^	0.86	2.34	2.874 (2)	121
O19—H191⋯O21	0.86	2.48	3.270 (4)	154
O19—H192⋯O2	0.86	1.99	2.830 (3)	166
O20—H201⋯O21	0.86	1.87	2.716 (3)	166
O20—H202⋯O17*A*^iii^	0.86	1.93	2.757 (2)	162
O21—H211⋯O1*A*	0.86	1.93	2.784 (3)	169
O21—H212⋯O18*B*^iii^	0.86	2.44	2.992 (3)	123

Symmetry codes: (i) 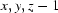; (ii) 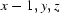; (iii) 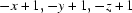; (iv) 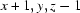; (v) 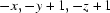; (vi) 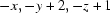; (vii) 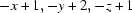; (viii) 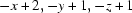; (ix) 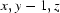; (x) 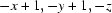.

**Fig. 1.**
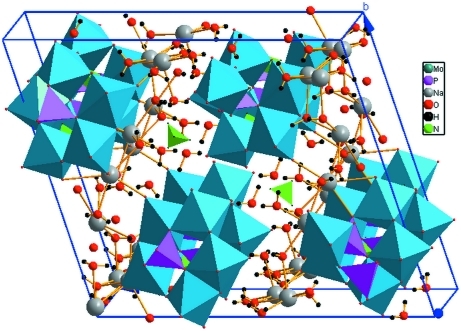
Polyhedral representation of the crystal packing of (NH4)5Na7[Mo5P2O23]2.21(H2O), viewed along the a direction.

**Fig. 2.**
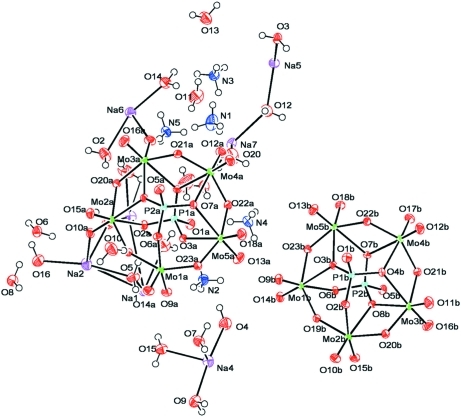
: Plot of the asymmetric unit of the title compound, showing the two independent [Mo5P2O23]6- anions with the same chirality and atom labeling scheme. Displacement ellipsoids are drawn at the 50% probability level.

**Fig. 3.**
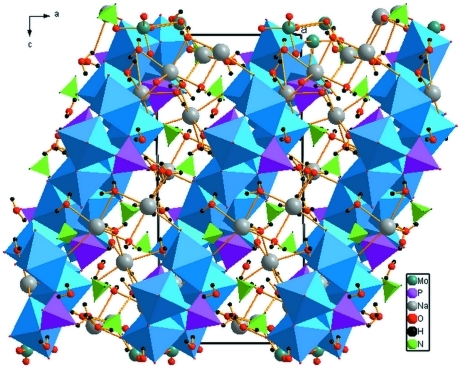
Projection of the structure of the title compound along the b axis, showing the layered arrangement of the structure parallel to the (101) plane.

**Fig. 4.**
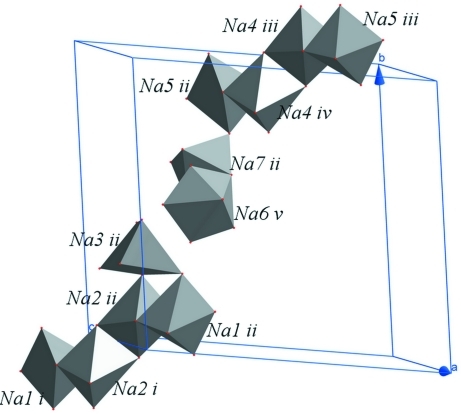
Interconnections of sodium octahedra in the crystal structure. Symetrie codes:i(x, -1+y, 1+z); ii(1-x,1-y, 1-z); iii(1+x, 1+y, z) iv(1-x, 2-y, 1-z); v(2-x, 1-y, 1-z)

## supplementary crystallographic information

### Comment

Among the numerous molybdenum phosphates that are actually known, those containing ammonium cations are of particular interest, since they are susceptible to being used as matrices to generate new phases in the Mo–P–O system without foreign cations, by extracting the ammonium ion using soft chemistry methods or electrochemistry. The number of ammonium polyoxomolybophosphates that are actually known is quite limited and some of them are cited here:(NH_4_)_3_[Mo_12_PO_40_].3(H_2_O) (Ferrari and Nanni, 1939), (NH_4_)_1.8_K_1.2_[Mo_12_PO_40_] (Boeyens *et al.*,1976), (NH_4_)_3_[Mo_12_PO_40_].21(H_2_O) (Xu *et al.*,1998), (NH_4_)_3_[Mo_12_PO_40_].*x*(H_2_O) (Ilhan *et al.* 2007) and (NH_4_)_8_Ni(HPO_4_)_2_[Mo_10_P_2_O_38_].12(H_2_O), (Andersen and Villadsen, 1993). We have thus revisited the system NH4–Na–P–Mo—O using slow evaporation synthesis. We have obtained a new mixed NH4—Na molybdenum (VI) phosphate hydrate corresponding to the chemical formula (NH_4_)_5_Na_7_[Mo_5_P_2_O_23_]_2_.21(H_2_O). Here we report on its structure.

A three-dimensional view of the structure is represented in Fig. 1. It shows that the structure is formed by almost regular PO4 tetrahedra linked to distorted oxygen octahedra around the Mo^vi+^ ions. The unit cell contains 4 [Mo_5_P_2_O_23_]^6-^ anions. Each anion is built up of five MoO6 octahedra sharing an edge and forming a ring which is closed by common corners of the terminal octahedra. The rings are closed on both sides by two asymmetric PO_4_ tetrahedra sharing three corners with three MoO_6_ octhedra as shown in Fig.2. Thus there are 2 independent anions with the same chirality in the asymmetric unit as shown in Fig. 2. In the crystal, the two enantiomers coexist.

The projection of the structure of the title compound along b direction (Fig.3), showing the layered arrangement of the [Mo_5_P_2_O_23_]^6-^ anions parallel to the plane (1 0 1). The layers are connected by two small chains of five octahedra of oxygen, more or less distorted, surrounding the sodium atoms. Besides, all sodium has an octahedral coordination number except Na6 who is in a bipyramid with a pentagonal basis. Each of the two chains is formed by 4 octahedra linked in pairs by an edge, while the fifth shares two vertices with the last two as shown in Fig. 4. It also shows that the second chain ends with a bipyramid-NaO7. Water molecules and Ammonium ions fill the remaining space and ensure the cohesion of the whole through the ionic and hydrogen bonds.

Heteropolyoxometallates are polyanions of general chemical formula [X_n_M_p_O_q_]^z-^. Their structures are characterized and distinguished by the form of their anionic blocks.  Among them, the structures with the Keggin anions [PMo_12_O_40_]^3-^ (12 MoO_6_ octahedra surrounding PO_4_ tetrahedron) are the most extensively investigated (Pope, 1983), followed by structures with heteropolyoxoanion [P_2_ Mo_5_O_23_]^6-^ called Strandberg structure (Strandberg, 1973; Hedman and Strandberg, 1979; Long *et al.* 2007). All these structures are built up by porous layers able to receive different organic ligands thus forming hybrid compounds (Ma *et al.* 2006; Wu et *al.*2009).

### Experimental

Colourless crystals of (NH_4_)_5_Na_7_[Mo_5_P_2_O_23_]_2_.21(H_2_O) were easily grown by slow evaporation at room temperature from aqueous solution of disodium molybdate dihydrate (Na_2_MoO_4_.2H_2_O) and ammonium phosphate (NH_4_H_2_PO_4_) with 1:1 molar ratio. The product was filtered off and washed with a mixture of ethanol/water (80/20).

### Refinement

All O-bound and N-bound H atoms were initially located in a difference map and refined with a O–H and N–H distance restraint of 0.84 (1) Å and 0.89 (1) Å respctively. An additional H···H restraint of 1.37 (2) Å and 1.44 (2) Åfor the water molecules and the ammonium respectively. Later they were refined in the ridingmodel with U_iso_(H) set to 1.2 U_eq_(O) or (N). The not significants bonds and angles were removed from the CIF file.

### Crystal data

**Table:**

(NH_4_)_5_Na_7_[Mo_5_P_2_O_23_]_2_·21H_2_O	*Z* = 2
*M**_r_* = 2448.76	*F*(000) = 2380
Triclinic, *P*1	*D*_x_ = 2.606 Mg m^−^^3^
Hall symbol: -p 1	Mo *K*α radiation, λ = 0.71073 Å
*a* = 9.2299 (3) Å	Cell parameters from 33624 reflections
*b* = 18.3516 (6) Å	θ = 1.4–38.0°
*c* = 19.7918 (6) Å	µ = 2.23 mm^−^^1^
α = 73.860 (1)°	*T* = 298 K
β = 85.323 (3)°	Paralellipiped, colourless
γ = 75.772 (2)°	0.42 × 0.14 × 0.08 mm
*V* = 3121.17 (17) Å^3^	

### Data collection

**Table:**

Bruker X8 APEXII Diffractometer	33626 independent reflections
Radiation source: fine-focus sealed tube	28095 reflections with *I* > 2σ(*I*)
graphite	*R*_int_ = 0.024
φ and ω scans	θ_max_ = 38.0°, θ_min_ = 1.4°
Absorption correction: multi-scan (*SADABS*; Bruker, 2005)	*h* = −15→15
*T*_min_ = 0.695, *T*_max_ = 0.837	*k* = −31→31
132450 measured reflections	*l* = −34→34

### Refinement

**Table:**

Refinement on *F*^2^	Secondary atom site location: difference Fourier map
Least-squares matrix: full	Hydrogen site location: inferred from neighbouring sites
*R*[*F*^2^ > 2σ(*F*^2^)] = 0.022	H-atom parameters constrained
*wR*(*F*^2^) = 0.057	*w* = 1/[σ^2^(*F*_o_^2^) + (0.0181*P*)^2^ + 2.3233*P*] where *P* = (*F*_o_^2^ + 2*F*_c_^2^)/3
*S* = 1.09	(Δ/σ)_max_ = 0.005
33626 reflections	Δρ_max_ = 0.99 e Å^−^^3^
839 parameters	Δρ_min_ = −0.76 e Å^−^^3^
0 restraints	Extinction correction: *SHELXL*, Fc^*^=kFc[1+0.001xFc^2^λ^3^/sin(2θ)]^-1/4^
Primary atom site location: structure-invariant direct methods	Extinction coefficient: 0.00095 (3)

### Special details

**Table:**

Geometry. All esds (except the esd in the dihedral angle between two l.s. planes) are estimated using the full covariance matrix. The cell esds are taken into account individually in the estimation of esds in distances, angles and torsion angles; correlations between esds in cell parameters are only used when they are defined by crystal symmetry. An approximate (isotropic) treatment of cell esds is used for estimating esds involving l.s. planes.
Refinement. Refinement of *F*^2^ against ALL reflections. The weighted *R*-factor wR and goodness of fit *S* are based on *F*^2^, conventional *R*-factors *R* are based on *F*, with *F* set to zero for negative *F*^2^. The threshold expression of *F*^2^ > σ(*F*^2^) is used only for calculating *R*-factors(gt) etc. and is not relevant to the choice of reflections for refinement. *R*-factors based on *F*^2^ are statistically about twice as large as those based on *F*, and *R*- factors based on ALL data will be even larger.

### Fractional atomic coordinates and isotropic or equivalent isotropic displacement parameters (Å^2^)

**Table:**

	*x*	*y*	*z*	*U*_iso_*/*U*_eq_	
Mo1A	0.492310 (12)	0.902494 (7)	0.343660 (6)	0.01467 (2)	
Mo2A	0.735113 (12)	0.814966 (7)	0.215956 (6)	0.01368 (2)	
Mo3A	0.873090 (13)	0.628793 (7)	0.303128 (6)	0.01678 (2)	
Mo4A	0.678462 (13)	0.587156 (7)	0.453493 (6)	0.01620 (2)	
Mo5A	0.433389 (12)	0.748439 (7)	0.472935 (6)	0.01530 (2)	
P1A	0.48960 (4)	0.71885 (2)	0.308740 (17)	0.01286 (5)	
P2A	0.79211 (3)	0.75969 (2)	0.402960 (17)	0.01274 (5)	
O1A	0.34812 (11)	0.69257 (7)	0.30366 (6)	0.02106 (19)	
O2A	0.53301 (11)	0.76604 (6)	0.23641 (5)	0.01649 (17)	
O3A	0.46154 (11)	0.77456 (6)	0.35793 (5)	0.01569 (16)	
O4A	0.62074 (11)	0.64826 (6)	0.33780 (5)	0.01611 (16)	
O5A	0.94463 (11)	0.74016 (7)	0.43521 (6)	0.0215 (2)	
O6A	0.71593 (11)	0.84625 (6)	0.39166 (5)	0.01732 (17)	
O7A	0.68843 (11)	0.71080 (6)	0.45080 (5)	0.01592 (16)	
O8A	0.80787 (11)	0.74280 (6)	0.32905 (5)	0.01586 (16)	
O9A	0.31315 (12)	0.93039 (7)	0.31154 (6)	0.0243 (2)	
O10A	0.67181 (12)	0.85621 (7)	0.13130 (5)	0.02108 (19)	
O11A	0.84747 (16)	0.55099 (7)	0.27801 (7)	0.0302 (3)	
O12A	0.64223 (15)	0.50469 (7)	0.44157 (7)	0.0278 (2)	
O13A	0.24416 (13)	0.75961 (8)	0.47347 (7)	0.0309 (3)	
O14A	0.52178 (14)	0.98849 (7)	0.35016 (7)	0.0258 (2)	
O15A	0.89771 (12)	0.84629 (7)	0.21162 (6)	0.02135 (19)	
O16A	1.06197 (13)	0.62069 (9)	0.29542 (7)	0.0341 (3)	
O17A	0.74864 (14)	0.55493 (7)	0.53758 (6)	0.0265 (2)	
O18A	0.47398 (15)	0.73386 (8)	0.55946 (6)	0.0274 (2)	
O19A	0.60715 (11)	0.88732 (6)	0.26208 (5)	0.01796 (17)	
O20A	0.81587 (12)	0.70838 (6)	0.21550 (5)	0.01797 (17)	
O21A	0.86607 (11)	0.58826 (6)	0.40407 (5)	0.01919 (18)	
O22A	0.47846 (11)	0.64131 (6)	0.46786 (6)	0.01934 (18)	
O23A	0.44199 (12)	0.85665 (6)	0.44104 (5)	0.01914 (18)	
Mo1B	0.215242 (12)	0.815178 (6)	0.718288 (5)	0.01292 (2)	
Mo2B	−0.025676 (12)	0.907008 (6)	0.843473 (6)	0.01376 (2)	
Mo3B	−0.081624 (12)	0.756868 (7)	0.978050 (6)	0.01538 (2)	
Mo4B	0.167731 (12)	0.593177 (7)	0.963923 (6)	0.01502 (2)	
Mo5B	0.362696 (11)	0.634503 (7)	0.813014 (6)	0.01370 (2)	
P1B	0.27703 (3)	0.76807 (2)	0.906219 (17)	0.01229 (5)	
P2B	−0.02535 (3)	0.719500 (19)	0.817922 (17)	0.01141 (5)	
O1B	0.42849 (11)	0.75117 (7)	0.93786 (6)	0.02107 (19)	
O2B	0.19706 (11)	0.85435 (6)	0.89184 (5)	0.01634 (16)	
O3B	0.28897 (11)	0.74801 (6)	0.83384 (5)	0.01464 (16)	
O4B	0.17722 (11)	0.71920 (6)	0.95805 (5)	0.01549 (16)	
O5B	−0.16688 (11)	0.69176 (6)	0.81884 (5)	0.01820 (18)	
O6B	0.01321 (10)	0.76426 (6)	0.74363 (5)	0.01486 (16)	
O7B	0.10810 (10)	0.64902 (6)	0.84613 (5)	0.01464 (15)	
O8B	−0.04676 (10)	0.77750 (6)	0.86459 (5)	0.01455 (15)	
O9B	0.37818 (12)	0.84669 (7)	0.71131 (6)	0.02057 (19)	
O10B	0.00007 (13)	0.99519 (6)	0.84546 (6)	0.0247 (2)	
O11B	−0.03688 (16)	0.74633 (8)	1.06283 (6)	0.0306 (3)	
O12B	0.23471 (14)	0.56392 (8)	1.04855 (6)	0.0270 (2)	
O13B	0.54923 (12)	0.63231 (7)	0.80612 (6)	0.0250 (2)	
O14B	0.15290 (12)	0.85003 (7)	0.63305 (5)	0.02072 (19)	
O15B	−0.20435 (12)	0.93135 (7)	0.81118 (6)	0.0230 (2)	
O16B	−0.27055 (13)	0.76566 (8)	0.98036 (7)	0.0306 (3)	
O17B	0.13950 (14)	0.50961 (7)	0.95064 (6)	0.0253 (2)	
O18B	0.34637 (12)	0.55192 (6)	0.79159 (6)	0.0218 (2)	
O19B	0.08888 (11)	0.88917 (6)	0.76195 (5)	0.01694 (17)	
O20B	−0.07788 (12)	0.86570 (6)	0.94177 (5)	0.01857 (18)	
O21B	−0.03213 (11)	0.64729 (6)	0.97706 (5)	0.01781 (17)	
O22B	0.35501 (11)	0.59687 (6)	0.91380 (5)	0.01754 (17)	
O23B	0.29709 (11)	0.70706 (6)	0.72405 (5)	0.01634 (16)	
Na1	0.09393 (8)	0.88130 (4)	0.12276 (4)	0.02741 (13)	
Na2	0.44879 (8)	0.90873 (5)	0.05715 (4)	0.03205 (15)	
Na3	0.32566 (11)	0.69247 (6)	0.06676 (5)	0.0475 (2)	
Na4	0.06704 (8)	1.08630 (5)	0.44343 (4)	0.03092 (15)	
Na5	0.42461 (8)	0.11186 (4)	0.37681 (4)	0.02557 (13)	
Na6	0.89731 (8)	0.47633 (4)	0.19135 (4)	0.02833 (14)	
Na7	0.23473 (7)	0.36371 (4)	0.26312 (4)	0.02572 (13)	
O1	0.13802 (15)	0.50097 (8)	0.18793 (7)	0.0349 (3)	
H11	0.1883	0.5175	0.1507	0.042*	
H12	0.1380	0.5319	0.2135	0.042*	
O2	0.80373 (18)	0.61051 (8)	0.12368 (7)	0.0367 (3)	
H21	0.8552	0.6219	0.0856	0.044*	
H22	0.8029	0.6464	0.1440	0.044*	
O3	0.62913 (13)	0.07746 (7)	0.45018 (6)	0.0247 (2)	
H31	0.6022	0.1008	0.4829	0.030*	
H32	0.7062	0.0937	0.4306	0.030*	
O4	0.07634 (18)	0.95082 (9)	0.44777 (8)	0.0388 (3)	
H41	0.0226	0.9483	0.4154	0.047*	
H42	0.1613	0.9197	0.4441	0.047*	
O5	0.30931 (14)	0.89540 (8)	0.17057 (7)	0.0305 (3)	
H51	0.2935	0.9324	0.1910	0.037*	
H52	0.3727	0.8577	0.1967	0.037*	
O6	0.88411 (14)	0.93215 (7)	0.04955 (6)	0.0255 (2)	
H61	0.8036	0.9204	0.0696	0.031*	
H62	0.9007	0.9106	0.0154	0.031*	
O7	0.29608 (14)	1.08048 (8)	0.49264 (6)	0.0285 (2)	
H71	0.3299	1.0322	0.5140	0.034*	
H72	0.2947	1.1052	0.5238	0.034*	
O8	0.77938 (14)	1.08752 (8)	−0.00967 (6)	0.0284 (2)	
H81	0.8193	1.0390	0.0084	0.034*	
H82	0.7783	1.1094	0.0237	0.034*	
O9	0.02918 (15)	1.22207 (8)	0.44210 (7)	0.0340 (3)	
H91	0.0378	1.2286	0.4828	0.041*	
H92	−0.0655	1.2310	0.4362	0.041*	
O10	0.89823 (18)	0.95851 (9)	0.34047 (9)	0.0431 (3)	
H101	0.8709	1.0015	0.3083	0.052*	
H102	0.8217	0.9383	0.3480	0.052*	
O11	0.97177 (19)	0.40736 (11)	0.43052 (9)	0.0483 (4)	
H111	1.0492	0.3742	0.4508	0.058*	
H112	0.9798	0.4515	0.4348	0.058*	
O12	0.29779 (15)	0.24836 (8)	0.36313 (7)	0.0340 (3)	
H121	0.2204	0.2434	0.3897	0.041*	
H122	0.3532	0.2646	0.3859	0.041*	
O13	0.60970 (18)	0.03813 (9)	0.15981 (9)	0.0421 (3)	
H131	0.6343	−0.0059	0.1909	0.051*	
H132	0.6907	0.0544	0.1504	0.051*	
O14	0.97224 (15)	0.37508 (8)	0.30176 (7)	0.0306 (3)	
H141	0.9733	0.3798	0.3437	0.037*	
H142	0.9575	0.3293	0.3072	0.037*	
O15	0.20367 (14)	1.10295 (8)	0.32985 (7)	0.0295 (2)	
H151	0.1455	1.1423	0.3027	0.035*	
H152	0.2219	1.0672	0.3079	0.035*	
O16	0.57481 (17)	0.95301 (9)	−0.05286 (8)	0.0368 (3)	
H161	0.5189	0.9503	−0.0844	0.044*	
H162	0.6604	0.9235	−0.0586	0.044*	
O17	0.49009 (18)	0.76434 (10)	0.08862 (9)	0.0459 (4)	
H171	0.4343	0.7740	0.0530	0.055*	
H172	0.5799	0.7584	0.0719	0.055*	
O18	0.18027 (19)	0.73673 (10)	0.16301 (10)	0.0514 (4)	
H181	0.2314	0.7056	0.1984	0.062*	
H182	0.1004	0.7201	0.1644	0.062*	
O19	0.5309 (2)	0.58788 (10)	0.08724 (11)	0.0571 (5)	
H191	0.4706	0.5935	0.1219	0.069*	
H192	0.6172	0.5864	0.1018	0.069*	
O20	0.3219 (2)	0.43251 (11)	0.32741 (9)	0.0601 (5)	
H201	0.3317	0.4783	0.3045	0.072*	
H202	0.2804	0.4380	0.3668	0.072*	
O21	0.3970 (3)	0.56485 (13)	0.24794 (15)	0.0976 (10)	
H211	0.3734	0.6074	0.2608	0.117*	
H212	0.4884	0.5445	0.2604	0.117*	
N1	0.6411 (2)	0.40411 (12)	0.36141 (10)	0.0471 (5)	
H11N	0.6534	0.4101	0.4023	0.057*	
H12N	0.5495	0.4016	0.3579	0.057*	
H13N	0.6600	0.4434	0.3288	0.057*	
H14N	0.7013	0.3614	0.3566	0.057*	
N2	0.11305 (15)	0.81900 (9)	0.32080 (8)	0.0282 (3)	
H21N	0.0788	0.8036	0.3631	0.034*	
H22N	0.1515	0.8585	0.3177	0.034*	
H23N	0.1812	0.7812	0.3112	0.034*	
H24N	0.0408	0.8327	0.2912	0.034*	
N3	0.40355 (15)	0.17946 (9)	0.17830 (8)	0.0272 (3)	
H31N	0.3325	0.2178	0.1847	0.033*	
H32N	0.4735	0.1694	0.2085	0.033*	
H33N	0.4400	0.1921	0.1360	0.033*	
H34N	0.3682	0.1385	0.1839	0.033*	
N4	0.20062 (16)	0.61691 (8)	0.42575 (8)	0.0266 (3)	
H41N	0.2412	0.6370	0.3861	0.032*	
H42N	0.1203	0.6498	0.4335	0.032*	
H43N	0.2631	0.6064	0.4595	0.032*	
H44N	0.1779	0.5744	0.4240	0.032*	
N5	0.32188 (16)	0.37776 (8)	0.06106 (7)	0.0258 (3)	
H51N	0.3937	0.3413	0.0517	0.031*	
H52N	0.2747	0.3584	0.0989	0.031*	
H53N	0.3587	0.4145	0.0674	0.031*	
H54N	0.2603	0.3968	0.0263	0.031*	

### Atomic displacement parameters (Å^2^)

**Table:**

	*U*^11^	*U*^22^	*U*^33^	*U*^12^	*U*^13^	*U*^23^
Mo1A	0.01617 (4)	0.01225 (5)	0.01538 (5)	−0.00185 (3)	0.00054 (3)	−0.00482 (4)
Mo2A	0.01413 (4)	0.01481 (5)	0.01165 (4)	−0.00409 (3)	0.00129 (3)	−0.00259 (3)
Mo3A	0.01754 (5)	0.01578 (5)	0.01542 (5)	−0.00018 (4)	0.00213 (3)	−0.00550 (4)
Mo4A	0.02082 (5)	0.01240 (5)	0.01418 (5)	−0.00345 (4)	0.00126 (4)	−0.00243 (4)
Mo5A	0.01518 (4)	0.01755 (5)	0.01396 (4)	−0.00465 (4)	0.00283 (3)	−0.00563 (4)
P1A	0.01306 (12)	0.01411 (14)	0.01280 (13)	−0.00474 (10)	0.00051 (10)	−0.00464 (11)
P2A	0.01218 (12)	0.01468 (14)	0.01270 (13)	−0.00422 (10)	0.00020 (9)	−0.00498 (11)
O1A	0.0167 (4)	0.0261 (5)	0.0247 (5)	−0.0107 (4)	0.0004 (3)	−0.0089 (4)
O2A	0.0171 (4)	0.0202 (5)	0.0126 (4)	−0.0065 (3)	−0.0001 (3)	−0.0033 (3)
O3A	0.0193 (4)	0.0145 (4)	0.0138 (4)	−0.0031 (3)	0.0007 (3)	−0.0056 (3)
O4A	0.0179 (4)	0.0138 (4)	0.0162 (4)	−0.0022 (3)	−0.0004 (3)	−0.0044 (3)
O5A	0.0142 (4)	0.0290 (6)	0.0231 (5)	−0.0043 (4)	−0.0033 (3)	−0.0095 (4)
O6A	0.0192 (4)	0.0148 (4)	0.0200 (4)	−0.0051 (3)	−0.0006 (3)	−0.0069 (4)
O7A	0.0166 (4)	0.0164 (4)	0.0155 (4)	−0.0057 (3)	0.0021 (3)	−0.0044 (3)
O8A	0.0194 (4)	0.0160 (4)	0.0125 (4)	−0.0046 (3)	0.0015 (3)	−0.0043 (3)
O9A	0.0199 (5)	0.0229 (5)	0.0268 (5)	−0.0003 (4)	−0.0035 (4)	−0.0046 (4)
O10A	0.0214 (4)	0.0247 (5)	0.0150 (4)	−0.0054 (4)	−0.0010 (3)	−0.0018 (4)
O11A	0.0471 (7)	0.0181 (5)	0.0256 (6)	−0.0024 (5)	0.0010 (5)	−0.0110 (4)
O12A	0.0383 (6)	0.0181 (5)	0.0293 (6)	−0.0105 (5)	0.0022 (5)	−0.0070 (4)
O13A	0.0174 (5)	0.0394 (7)	0.0396 (7)	−0.0084 (5)	0.0059 (4)	−0.0166 (6)
O14A	0.0314 (6)	0.0168 (5)	0.0315 (6)	−0.0056 (4)	−0.0001 (4)	−0.0104 (4)
O15A	0.0196 (4)	0.0250 (5)	0.0198 (5)	−0.0102 (4)	0.0009 (3)	−0.0025 (4)
O16A	0.0188 (5)	0.0474 (8)	0.0289 (6)	−0.0007 (5)	0.0024 (4)	−0.0053 (6)
O17A	0.0339 (6)	0.0248 (6)	0.0170 (5)	−0.0046 (5)	−0.0014 (4)	−0.0011 (4)
O18A	0.0361 (6)	0.0311 (6)	0.0155 (5)	−0.0091 (5)	0.0012 (4)	−0.0064 (4)
O19A	0.0217 (4)	0.0148 (4)	0.0157 (4)	−0.0028 (3)	0.0030 (3)	−0.0037 (3)
O20A	0.0232 (4)	0.0171 (4)	0.0133 (4)	−0.0023 (3)	0.0016 (3)	−0.0062 (3)
O21A	0.0185 (4)	0.0187 (5)	0.0167 (4)	0.0002 (3)	0.0003 (3)	−0.0028 (4)
O22A	0.0199 (4)	0.0170 (4)	0.0222 (5)	−0.0076 (3)	0.0038 (3)	−0.0051 (4)
O23A	0.0268 (5)	0.0158 (4)	0.0156 (4)	−0.0046 (4)	0.0033 (3)	−0.0067 (3)
Mo1B	0.01374 (4)	0.01404 (5)	0.01068 (4)	−0.00449 (3)	0.00104 (3)	−0.00208 (3)
Mo2B	0.01503 (4)	0.01176 (4)	0.01424 (4)	−0.00222 (3)	0.00020 (3)	−0.00397 (4)
Mo3B	0.01626 (4)	0.01601 (5)	0.01367 (4)	−0.00422 (4)	0.00364 (3)	−0.00431 (4)
Mo4B	0.01617 (4)	0.01305 (5)	0.01367 (4)	−0.00304 (3)	0.00121 (3)	−0.00079 (4)
Mo5B	0.01213 (4)	0.01431 (5)	0.01421 (4)	−0.00206 (3)	0.00130 (3)	−0.00441 (4)
P1B	0.01225 (12)	0.01427 (14)	0.01123 (12)	−0.00434 (10)	−0.00025 (9)	−0.00368 (10)
P2B	0.01083 (11)	0.01214 (13)	0.01187 (12)	−0.00324 (9)	−0.00013 (9)	−0.00371 (10)
O1B	0.0157 (4)	0.0263 (5)	0.0218 (5)	−0.0053 (4)	−0.0049 (3)	−0.0058 (4)
O2B	0.0184 (4)	0.0146 (4)	0.0176 (4)	−0.0045 (3)	−0.0015 (3)	−0.0058 (3)
O3B	0.0181 (4)	0.0143 (4)	0.0118 (4)	−0.0043 (3)	0.0009 (3)	−0.0037 (3)
O4B	0.0166 (4)	0.0171 (4)	0.0128 (4)	−0.0063 (3)	0.0018 (3)	−0.0025 (3)
O5B	0.0145 (4)	0.0231 (5)	0.0200 (4)	−0.0089 (3)	0.0009 (3)	−0.0069 (4)
O6B	0.0154 (4)	0.0169 (4)	0.0120 (4)	−0.0049 (3)	−0.0003 (3)	−0.0025 (3)
O7B	0.0144 (4)	0.0127 (4)	0.0153 (4)	−0.0011 (3)	−0.0011 (3)	−0.0027 (3)
O8B	0.0174 (4)	0.0133 (4)	0.0135 (4)	−0.0031 (3)	0.0007 (3)	−0.0051 (3)
O9B	0.0189 (4)	0.0243 (5)	0.0195 (4)	−0.0103 (4)	0.0012 (3)	−0.0032 (4)
O10B	0.0299 (5)	0.0158 (5)	0.0304 (6)	−0.0066 (4)	−0.0001 (4)	−0.0085 (4)
O11B	0.0443 (7)	0.0324 (7)	0.0157 (5)	−0.0103 (5)	0.0027 (5)	−0.0068 (5)
O12B	0.0278 (5)	0.0321 (6)	0.0162 (5)	−0.0062 (5)	−0.0015 (4)	0.0013 (4)
O13B	0.0148 (4)	0.0357 (6)	0.0240 (5)	−0.0054 (4)	0.0019 (4)	−0.0083 (5)
O14B	0.0220 (4)	0.0257 (5)	0.0133 (4)	−0.0069 (4)	−0.0010 (3)	−0.0019 (4)
O15B	0.0175 (4)	0.0224 (5)	0.0260 (5)	−0.0014 (4)	−0.0036 (4)	−0.0036 (4)
O16B	0.0188 (5)	0.0315 (6)	0.0421 (7)	−0.0072 (4)	0.0080 (4)	−0.0120 (5)
O17B	0.0292 (5)	0.0160 (5)	0.0300 (6)	−0.0068 (4)	0.0020 (4)	−0.0043 (4)
O18B	0.0248 (5)	0.0169 (5)	0.0245 (5)	−0.0038 (4)	0.0014 (4)	−0.0082 (4)
O19B	0.0202 (4)	0.0146 (4)	0.0146 (4)	−0.0028 (3)	0.0022 (3)	−0.0035 (3)
O20B	0.0258 (5)	0.0164 (4)	0.0146 (4)	−0.0052 (4)	0.0037 (3)	−0.0069 (3)
O21B	0.0171 (4)	0.0158 (4)	0.0201 (4)	−0.0059 (3)	0.0033 (3)	−0.0034 (4)
O22B	0.0146 (4)	0.0198 (5)	0.0145 (4)	−0.0013 (3)	−0.0003 (3)	−0.0009 (3)
O23B	0.0193 (4)	0.0162 (4)	0.0131 (4)	−0.0026 (3)	0.0011 (3)	−0.0050 (3)
Na1	0.0274 (3)	0.0300 (4)	0.0237 (3)	−0.0067 (3)	−0.0013 (2)	−0.0051 (3)
Na2	0.0297 (3)	0.0363 (4)	0.0286 (4)	−0.0107 (3)	−0.0032 (3)	−0.0027 (3)
Na3	0.0512 (5)	0.0525 (6)	0.0403 (5)	−0.0036 (4)	−0.0161 (4)	−0.0176 (4)
Na4	0.0302 (3)	0.0353 (4)	0.0289 (3)	−0.0120 (3)	−0.0027 (3)	−0.0066 (3)
Na5	0.0250 (3)	0.0253 (3)	0.0259 (3)	−0.0044 (2)	−0.0026 (2)	−0.0068 (3)
Na6	0.0271 (3)	0.0251 (3)	0.0338 (4)	−0.0079 (3)	−0.0004 (3)	−0.0079 (3)
Na7	0.0231 (3)	0.0333 (4)	0.0241 (3)	−0.0099 (3)	0.0031 (2)	−0.0111 (3)
O1	0.0336 (6)	0.0361 (7)	0.0334 (7)	−0.0115 (5)	0.0041 (5)	−0.0048 (6)
O2	0.0540 (9)	0.0301 (7)	0.0288 (6)	−0.0174 (6)	−0.0055 (6)	−0.0046 (5)
O3	0.0302 (5)	0.0270 (6)	0.0200 (5)	−0.0103 (4)	0.0042 (4)	−0.0096 (4)
O4	0.0427 (8)	0.0394 (8)	0.0372 (7)	−0.0099 (6)	0.0035 (6)	−0.0160 (6)
O5	0.0268 (5)	0.0346 (7)	0.0283 (6)	0.0020 (5)	−0.0084 (4)	−0.0113 (5)
O6	0.0317 (6)	0.0285 (6)	0.0197 (5)	−0.0104 (5)	0.0033 (4)	−0.0098 (4)
O7	0.0337 (6)	0.0274 (6)	0.0234 (5)	−0.0048 (5)	−0.0051 (4)	−0.0059 (5)
O8	0.0343 (6)	0.0306 (6)	0.0218 (5)	−0.0065 (5)	−0.0026 (4)	−0.0098 (5)
O9	0.0326 (6)	0.0412 (8)	0.0309 (6)	−0.0082 (5)	0.0030 (5)	−0.0154 (6)
O10	0.0417 (8)	0.0362 (8)	0.0488 (9)	−0.0151 (6)	−0.0041 (7)	−0.0008 (7)
O11	0.0477 (9)	0.0604 (11)	0.0420 (9)	−0.0108 (8)	−0.0022 (7)	−0.0234 (8)
O12	0.0337 (6)	0.0326 (7)	0.0359 (7)	−0.0098 (5)	−0.0055 (5)	−0.0062 (6)
O13	0.0420 (8)	0.0310 (7)	0.0495 (9)	−0.0129 (6)	−0.0028 (7)	0.0000 (6)
O14	0.0402 (7)	0.0270 (6)	0.0267 (6)	−0.0076 (5)	−0.0010 (5)	−0.0107 (5)
O15	0.0270 (5)	0.0336 (7)	0.0271 (6)	0.0000 (5)	−0.0088 (4)	−0.0106 (5)
O16	0.0384 (7)	0.0374 (8)	0.0353 (7)	−0.0068 (6)	0.0061 (6)	−0.0145 (6)
O17	0.0406 (8)	0.0480 (9)	0.0589 (10)	−0.0150 (7)	0.0098 (7)	−0.0293 (8)
O18	0.0423 (9)	0.0435 (9)	0.0602 (11)	−0.0053 (7)	0.0079 (7)	−0.0070 (8)
O19	0.0437 (9)	0.0396 (9)	0.0789 (13)	−0.0083 (7)	−0.0148 (9)	0.0017 (9)
O20	0.0965 (15)	0.0632 (12)	0.0327 (8)	−0.0292 (11)	0.0098 (9)	−0.0261 (8)
O21	0.1028 (19)	0.0707 (15)	0.151 (2)	−0.0483 (14)	0.0766 (17)	−0.0777 (16)
N1	0.0635 (13)	0.0582 (12)	0.0340 (9)	−0.0283 (10)	0.0111 (8)	−0.0266 (9)
N2	0.0218 (6)	0.0331 (8)	0.0285 (7)	−0.0064 (5)	−0.0009 (5)	−0.0059 (6)
N3	0.0203 (5)	0.0284 (7)	0.0305 (7)	−0.0042 (5)	−0.0006 (5)	−0.0052 (6)
N4	0.0305 (6)	0.0221 (6)	0.0258 (6)	−0.0087 (5)	−0.0044 (5)	−0.0006 (5)
N5	0.0278 (6)	0.0243 (6)	0.0231 (6)	−0.0074 (5)	−0.0035 (5)	−0.0005 (5)

### Geometric parameters (Å, °)

**Table:**

Mo1A—O14A	1.7062 (11)	Na3—O19	2.311 (2)
Mo1A—O9A	1.7248 (11)	Na3—O17	2.3745 (18)
Mo1A—O19A	1.9063 (10)	Na3—O18	2.449 (2)
Mo1A—O23A	1.9449 (10)	Na3—O4B^ix^	2.5122 (13)
Mo1A—O6A	2.2261 (10)	Na3—O1B^ix^	2.6738 (15)
Mo1A—O3A	2.3703 (10)	Na3—O12B^ix^	2.8100 (16)
Mo2A—O10A	1.7200 (10)	Na4—O7	2.3657 (15)
Mo2A—O15A	1.7222 (10)	Na4—O9	2.4224 (16)
Mo2A—O20A	1.9166 (10)	Na4—O4	2.4442 (17)
Mo2A—O19A	1.9284 (10)	Na4—O4^vi^	2.4527 (17)
Mo2A—O2A	2.2243 (9)	Na4—O15	2.4661 (15)
Mo2A—O8A	2.3184 (10)	Na4—O14B^vi^	2.4757 (13)
Mo3A—O11A	1.7092 (12)	Na4—H92	2.6056
Mo3A—O16A	1.7107 (12)	Na5—O3	2.3286 (14)
Mo3A—O21A	1.9300 (10)	Na5—O15^x^	2.3677 (14)
Mo3A—O20A	1.9447 (10)	Na5—O14A^x^	2.4075 (13)
Mo3A—O8A	2.2204 (10)	Na5—O12	2.4396 (16)
Mo3A—O4A	2.3431 (10)	Na5—O7^x^	2.4805 (15)
Mo4A—O12A	1.7083 (11)	Na5—O9B^v^	2.5217 (12)
Mo4A—O17A	1.7260 (12)	Na6—O1^ii^	2.3659 (15)
Mo4A—O22A	1.9095 (11)	Na6—O18B^v^	2.4069 (13)
Mo4A—O21A	1.9198 (10)	Na6—O2	2.4316 (16)
Mo4A—O7A	2.2791 (10)	Na6—O14	2.4624 (15)
Mo4A—O4A	2.2939 (10)	Na6—O7B^v^	2.6182 (12)
Mo5A—O13A	1.7073 (11)	Na6—O17B^v^	2.7909 (15)
Mo5A—O18A	1.7161 (11)	Na7—O20	2.3281 (18)
Mo5A—O23A	1.9289 (10)	Na7—O13B^v^	2.3302 (12)
Mo5A—O22A	1.9362 (10)	Na7—O5B^iv^	2.3360 (12)
Mo5A—O3A	2.1992 (9)	Na7—O12	2.4476 (15)
Mo5A—O7A	2.3262 (10)	Na7—O14^vii^	2.4568 (15)
P1A—O1A	1.5185 (10)	Na7—O1	2.5266 (16)
P1A—O2A	1.5323 (10)	O1—Na6^vii^	2.3659 (15)
P1A—O4A	1.5499 (10)	O1—H11	0.8599
P1A—O3A	1.5629 (10)	O1—H12	0.8598
P2A—O5A	1.5129 (10)	O2—H21	0.8600
P2A—O6A	1.5319 (11)	O2—H22	0.8600
P2A—O7A	1.5554 (10)	O3—H31	0.8598
P2A—O8A	1.5660 (10)	O3—H32	0.8599
O10A—Na2	2.4599 (13)	O4—Na4^vi^	2.4527 (17)
O11A—Na6	2.4288 (14)	O4—H41	0.8599
O14A—Na5^i^	2.4075 (13)	O4—H42	0.8600
O15A—Na1^ii^	2.4920 (12)	O5—H51	0.8599
Mo1B—O14B	1.7214 (10)	O5—H52	0.8599
Mo1B—O9B	1.7217 (10)	O6—Na1^ii^	2.3534 (14)
Mo1B—O19B	1.9132 (10)	O6—H61	0.8600
Mo1B—O23B	1.9162 (10)	O6—H62	0.8599
Mo1B—O6B	2.2462 (9)	O7—Na5^i^	2.4806 (15)
Mo1B—O3B	2.3424 (9)	O7—H71	0.8597
Mo2B—O10B	1.7038 (11)	O7—H72	0.8599
Mo2B—O15B	1.7259 (11)	O8—Na2^viii^	2.3534 (15)
Mo2B—O19B	1.9168 (10)	O8—Na1^viii^	2.4279 (14)
Mo2B—O20B	1.9489 (10)	O8—H81	0.8600
Mo2B—O2B	2.2150 (10)	O8—H82	0.8600
Mo2B—O8B	2.3501 (10)	O9—H91	0.8597
Mo3B—O11B	1.7079 (12)	O9—H92	0.8598
Mo3B—O16B	1.7092 (12)	O10—H101	0.8598
Mo3B—O20B	1.9335 (10)	O10—H102	0.8599
Mo3B—O21B	1.9550 (10)	O11—H111	0.8598
Mo3B—O8B	2.1815 (9)	O11—H112	0.8597
Mo3B—O4B	2.3512 (10)	O12—H121	0.8598
Mo4B—O17B	1.7090 (11)	O12—H122	0.8598
Mo4B—O12B	1.7268 (11)	O13—H131	0.8599
Mo4B—O21B	1.9030 (10)	O13—H132	0.8598
Mo4B—O22B	1.9280 (10)	O14—Na7^ii^	2.4568 (15)
Mo4B—O4B	2.3062 (10)	O14—H141	0.8598
Mo4B—O7B	2.3221 (10)	O14—H142	0.8599
Mo5B—O13B	1.7074 (10)	O15—Na5^i^	2.3677 (14)
Mo5B—O18B	1.7261 (11)	O15—H151	0.8599
Mo5B—O22B	1.9225 (10)	O15—H152	0.8599
Mo5B—O23B	1.9272 (10)	O16—Na2^viii^	2.4707 (17)
Mo5B—O3B	2.1696 (10)	O16—H161	0.8599
Mo5B—O7B	2.3581 (9)	O16—H162	0.8598
P1B—O1B	1.5047 (10)	O17—H171	0.8598
P1B—O2B	1.5304 (10)	O17—H172	0.8600
P1B—O4B	1.5639 (10)	O18—H181	0.8599
P1B—O3B	1.5643 (10)	O18—H182	0.8599
P2B—O5B	1.5113 (10)	O19—H191	0.8599
P2B—O6B	1.5331 (10)	O19—H192	0.8600
P2B—O7B	1.5586 (10)	O20—H201	0.8600
P2B—O8B	1.5616 (10)	O20—H202	0.8600
O1B—Na3^iii^	2.6738 (15)	O21—H211	0.8601
O4B—Na3^iii^	2.5122 (13)	O21—H212	0.8601
O5B—Na7^iv^	2.3360 (12)	N1—H11N	0.8669
O7B—Na6^v^	2.6182 (12)	N1—H12N	0.8668
O9B—Na5^v^	2.5217 (12)	N1—H13N	0.8669
O10B—Na1^vi^	2.4541 (13)	N1—H14N	0.8668
O12B—Na3^iii^	2.8100 (16)	N2—H21N	0.8667
O13B—Na7^v^	2.3302 (12)	N2—H22N	0.8669
O14B—Na4^vi^	2.4758 (13)	N2—H23N	0.8668
O17B—Na6^v^	2.7909 (15)	N2—H24N	0.8669
O18B—Na6^v^	2.4069 (13)	N3—H31N	0.8670
Na1—O6^vii^	2.3534 (14)	N3—H32N	0.8670
Na1—O5	2.3686 (14)	N3—H33N	0.8668
Na1—O8^viii^	2.4279 (14)	N3—H34N	0.8667
Na1—O10B^vi^	2.4541 (13)	N4—H41N	0.8666
Na1—O18	2.4895 (19)	N4—H42N	0.8669
Na1—O15A^vii^	2.4920 (12)	N4—H43N	0.8669
Na2—O8^viii^	2.3533 (15)	N4—H44N	0.8670
Na2—O16	2.4129 (16)	N5—H51N	0.8667
Na2—O16^viii^	2.4707 (17)	N5—H52N	0.8672
Na2—O5	2.4768 (15)	N5—H53N	0.8669
Na2—O17	2.4858 (18)	N5—H54N	0.8666
			
O14A—Mo1A—O9A	102.22 (6)	O8^viii^—Na1—O15A^vii^	159.95 (5)
O14A—Mo1A—O19A	102.60 (5)	O10B^vi^—Na1—O15A^vii^	79.37 (4)
O9A—Mo1A—O19A	101.23 (5)	O18—Na1—O15A^vii^	78.15 (5)
O14A—Mo1A—O23A	100.23 (5)	O8^viii^—Na2—O16	97.26 (6)
O9A—Mo1A—O23A	97.14 (5)	O8^viii^—Na2—O10A	158.25 (6)
O19A—Mo1A—O23A	146.77 (4)	O16—Na2—O10A	97.36 (5)
O14A—Mo1A—O6A	86.85 (5)	O8^viii^—Na2—O16^viii^	102.28 (6)
O9A—Mo1A—O6A	170.21 (5)	O16—Na2—O16^viii^	78.87 (5)
O19A—Mo1A—O6A	80.08 (4)	O10A—Na2—O16^viii^	96.29 (5)
O23A—Mo1A—O6A	77.39 (4)	O8^viii^—Na2—O5	84.99 (5)
O14A—Mo1A—O3A	169.02 (5)	O16—Na2—O5	166.63 (6)
O9A—Mo1A—O3A	83.84 (5)	O10A—Na2—O5	84.51 (4)
O19A—Mo1A—O3A	84.95 (4)	O16^viii^—Na2—O5	87.78 (5)
O23A—Mo1A—O3A	69.70 (4)	O8^viii^—Na2—O17	86.32 (6)
O6A—Mo1A—O3A	86.62 (3)	O16—Na2—O17	109.59 (6)
O10A—Mo2A—O15A	101.45 (5)	O10A—Na2—O17	73.63 (5)
O10A—Mo2A—O20A	101.79 (5)	O16^viii^—Na2—O17	167.31 (6)
O15A—Mo2A—O20A	100.16 (5)	O5—Na2—O17	83.66 (5)
O10A—Mo2A—O19A	99.60 (5)	O19—Na3—O17	85.28 (7)
O15A—Mo2A—O19A	99.19 (5)	O19—Na3—O18	121.92 (8)
O20A—Mo2A—O19A	147.53 (4)	O17—Na3—O18	82.98 (6)
O10A—Mo2A—O2A	86.21 (4)	O19—Na3—O4B^ix^	117.80 (7)
O15A—Mo2A—O2A	172.34 (4)	O17—Na3—O4B^ix^	127.96 (7)
O20A—Mo2A—O2A	77.78 (4)	O18—Na3—O4B^ix^	113.98 (6)
O19A—Mo2A—O2A	79.58 (4)	O19—Na3—O1B^ix^	91.20 (7)
O10A—Mo2A—O8A	171.17 (5)	O17—Na3—O1B^ix^	77.46 (6)
O15A—Mo2A—O8A	85.41 (4)	O18—Na3—O1B^ix^	139.83 (6)
O20A—Mo2A—O8A	71.26 (4)	O4B^ix^—Na3—O1B^ix^	57.64 (4)
O19A—Mo2A—O8A	84.61 (4)	O19—Na3—O12B^ix^	72.69 (6)
O2A—Mo2A—O8A	86.94 (3)	O17—Na3—O12B^ix^	157.94 (6)
O11A—Mo3A—O16A	104.13 (7)	O18—Na3—O12B^ix^	109.41 (6)
O11A—Mo3A—O21A	100.05 (5)	O4B^ix^—Na3—O12B^ix^	64.75 (4)
O16A—Mo3A—O21A	96.78 (5)	O1B^ix^—Na3—O12B^ix^	101.35 (4)
O11A—Mo3A—O20A	97.90 (5)	O19—Na3—P1B^ix^	111.15 (6)
O16A—Mo3A—O20A	97.92 (6)	O17—Na3—P1B^ix^	99.82 (6)
O21A—Mo3A—O20A	153.22 (4)	O18—Na3—P1B^ix^	126.87 (6)
O11A—Mo3A—O8A	156.81 (5)	O4B^ix^—Na3—P1B^ix^	29.91 (2)
O16A—Mo3A—O8A	98.32 (6)	O1B^ix^—Na3—P1B^ix^	28.87 (2)
O21A—Mo3A—O8A	82.77 (4)	O12B^ix^—Na3—P1B^ix^	87.39 (3)
O20A—Mo3A—O8A	73.05 (4)	O19—Na3—Mo4B^ix^	88.18 (6)
O11A—Mo3A—O4A	86.34 (5)	O17—Na3—Mo4B^ix^	155.44 (6)
O16A—Mo3A—O4A	166.47 (5)	O18—Na3—Mo4B^ix^	120.22 (6)
O21A—Mo3A—O4A	72.64 (4)	O4B^ix^—Na3—Mo4B^ix^	38.41 (3)
O20A—Mo3A—O4A	88.92 (4)	O1B^ix^—Na3—Mo4B^ix^	79.04 (3)
O8A—Mo3A—O4A	72.44 (3)	O12B^ix^—Na3—Mo4B^ix^	27.02 (2)
O12A—Mo4A—O17A	102.25 (6)	P1B^ix^—Na3—Mo4B^ix^	60.775 (16)
O12A—Mo4A—O22A	98.92 (5)	O19—Na3—Na1	146.98 (6)
O17A—Mo4A—O22A	102.37 (5)	O17—Na3—Na1	69.69 (5)
O12A—Mo4A—O21A	100.92 (5)	O18—Na3—Na1	36.46 (4)
O17A—Mo4A—O21A	97.16 (5)	O4B^ix^—Na3—Na1	94.86 (4)
O22A—Mo4A—O21A	148.39 (4)	O1B^ix^—Na3—Na1	103.43 (4)
O12A—Mo4A—O7A	167.40 (5)	O12B^ix^—Na3—Na1	130.91 (4)
O17A—Mo4A—O7A	89.35 (5)	O19—Na3—H171	97.1
O22A—Mo4A—O7A	73.47 (4)	O17—Na3—H171	20.0
O21A—Mo4A—O7A	82.23 (4)	O18—Na3—H171	90.3
O12A—Mo4A—O4A	88.68 (5)	O4B^ix^—Na3—H171	108.0
O17A—Mo4A—O4A	167.20 (5)	O1B^ix^—Na3—H171	61.2
O22A—Mo4A—O4A	82.18 (4)	O12B^ix^—Na3—H171	160.3
O21A—Mo4A—O4A	73.97 (4)	O19—Na3—H191	21.7
O7A—Mo4A—O4A	80.42 (3)	O17—Na3—H191	87.4
O13A—Mo5A—O18A	103.91 (6)	O18—Na3—H191	100.9
O13A—Mo5A—O23A	99.20 (6)	O4B^ix^—Na3—H191	131.5
O18A—Mo5A—O23A	97.31 (5)	O1B^ix^—Na3—H191	112.7
O13A—Mo5A—O22A	94.56 (5)	O12B^ix^—Na3—H191	72.6
O18A—Mo5A—O22A	99.91 (5)	H171—Na3—H191	104.2
O23A—Mo5A—O22A	154.66 (4)	O7—Na4—O9	80.79 (5)
O13A—Mo5A—O3A	95.75 (5)	O7—Na4—O4	104.19 (6)
O18A—Mo5A—O3A	159.60 (5)	O9—Na4—O4	173.79 (6)
O23A—Mo5A—O3A	73.89 (4)	O7—Na4—O4^vi^	99.21 (6)
O22A—Mo5A—O3A	83.68 (4)	O9—Na4—O4^vi^	95.84 (5)
O13A—Mo5A—O7A	163.36 (5)	O4—Na4—O4^vi^	79.81 (6)
O18A—Mo5A—O7A	88.32 (5)	O7—Na4—O15	85.76 (5)
O23A—Mo5A—O7A	90.22 (4)	O9—Na4—O15	93.57 (5)
O22A—Mo5A—O7A	71.92 (4)	O4—Na4—O15	90.52 (5)
O3A—Mo5A—O7A	73.61 (4)	O4^vi^—Na4—O15	169.95 (6)
O1A—P1A—O2A	110.26 (6)	O7—Na4—O14B^vi^	154.61 (5)
O1A—P1A—O4A	111.26 (6)	O9—Na4—O14B^vi^	77.41 (5)
O2A—P1A—O4A	109.65 (6)	O4—Na4—O14B^vi^	98.51 (5)
O1A—P1A—O3A	109.98 (6)	O4^vi^—Na4—O14B^vi^	95.83 (5)
O2A—P1A—O3A	106.42 (6)	O15—Na4—O14B^vi^	82.78 (4)
O4A—P1A—O3A	109.15 (6)	O7—Na4—H92	99.3
O5A—P2A—O6A	111.48 (6)	O9—Na4—H92	19.2
O5A—P2A—O7A	110.97 (6)	O4—Na4—H92	154.8
O6A—P2A—O7A	108.23 (6)	O4^vi^—Na4—H92	87.6
O5A—P2A—O8A	109.93 (6)	O15—Na4—H92	100.2
O6A—P2A—O8A	107.03 (6)	O14B^vi^—Na4—H92	60.9
O7A—P2A—O8A	109.09 (5)	Na5^i^—Na4—H92	99.3
O14B—Mo1B—O9B	101.76 (5)	Na4^vi^—Na4—H92	124.2
O14B—Mo1B—O19B	101.50 (5)	O3—Na5—O15^x^	158.87 (6)
O9B—Mo1B—O19B	99.54 (5)	O3—Na5—O14A^x^	86.21 (5)
O14B—Mo1B—O23B	101.46 (5)	O15^x^—Na5—O14A^x^	82.36 (5)
O9B—Mo1B—O23B	99.65 (5)	O3—Na5—O12	110.20 (5)
O19B—Mo1B—O23B	146.22 (4)	O15^x^—Na5—O12	84.84 (5)
O14B—Mo1B—O6B	86.61 (4)	O14A^x^—Na5—O12	160.63 (5)
O9B—Mo1B—O6B	171.55 (4)	O3—Na5—O7^x^	80.56 (5)
O19B—Mo1B—O6B	79.67 (4)	O15^x^—Na5—O7^x^	85.39 (5)
O23B—Mo1B—O6B	77.31 (4)	O14A^x^—Na5—O7^x^	105.30 (5)
O14B—Mo1B—O3B	170.15 (4)	O12—Na5—O7^x^	88.03 (5)
O9B—Mo1B—O3B	85.40 (4)	O3—Na5—O9B^v^	79.66 (4)
O19B—Mo1B—O3B	83.76 (4)	O15^x^—Na5—O9B^v^	116.19 (5)
O23B—Mo1B—O3B	70.39 (4)	O14A^x^—Na5—O9B^v^	82.76 (4)
O6B—Mo1B—O3B	86.16 (3)	O12—Na5—O9B^v^	89.96 (5)
O10B—Mo2B—O15B	102.25 (6)	O7^x^—Na5—O9B^v^	158.06 (5)
O10B—Mo2B—O19B	102.34 (5)	O1^ii^—Na6—O18B^v^	173.83 (6)
O15B—Mo2B—O19B	100.28 (5)	O1^ii^—Na6—O11A	83.19 (5)
O10B—Mo2B—O20B	100.37 (5)	O18B^v^—Na6—O11A	92.36 (5)
O15B—Mo2B—O20B	96.72 (5)	O1^ii^—Na6—O2	89.19 (5)
O19B—Mo2B—O20B	147.90 (4)	O18B^v^—Na6—O2	93.83 (5)
O10B—Mo2B—O2B	87.44 (5)	O11A—Na6—O2	75.31 (5)
O15B—Mo2B—O2B	169.73 (5)	O1^ii^—Na6—O14	86.93 (5)
O19B—Mo2B—O2B	80.68 (4)	O18B^v^—Na6—O14	87.94 (5)
O20B—Mo2B—O2B	77.97 (4)	O11A—Na6—O14	77.56 (5)
O10B—Mo2B—O8B	168.43 (5)	O2—Na6—O14	152.86 (5)
O15B—Mo2B—O8B	84.88 (5)	O1^ii^—Na6—O7B^v^	114.27 (5)
O19B—Mo2B—O8B	85.09 (4)	O18B^v^—Na6—O7B^v^	68.03 (4)
O20B—Mo2B—O8B	69.48 (4)	O11A—Na6—O7B^v^	150.16 (5)
O2B—Mo2B—O8B	85.02 (3)	O2—Na6—O7B^v^	126.23 (5)
O11B—Mo3B—O16B	104.68 (7)	O14—Na6—O7B^v^	79.40 (4)
O11B—Mo3B—O20B	97.40 (5)	O1^ii^—Na6—O17B^v^	100.56 (5)
O16B—Mo3B—O20B	99.23 (6)	O18B^v^—Na6—O17B^v^	85.58 (4)
O11B—Mo3B—O21B	99.59 (6)	O11A—Na6—O17B^v^	142.03 (5)
O16B—Mo3B—O21B	94.53 (5)	O2—Na6—O17B^v^	67.03 (4)
O20B—Mo3B—O21B	154.64 (4)	O14—Na6—O17B^v^	140.04 (5)
O11B—Mo3B—O8B	156.61 (5)	O7B^v^—Na6—O17B^v^	61.63 (3)
O16B—Mo3B—O8B	98.14 (5)	O20—Na7—O13B^v^	87.38 (6)
O20B—Mo3B—O8B	73.56 (4)	O20—Na7—O5B^iv^	169.63 (6)
O21B—Mo3B—O8B	83.51 (4)	O13B^v^—Na7—O5B^iv^	84.19 (4)
O11B—Mo3B—O4B	85.93 (5)	O20—Na7—O12	87.87 (6)
O16B—Mo3B—O4B	164.14 (5)	O13B^v^—Na7—O12	106.61 (5)
O20B—Mo3B—O4B	90.88 (4)	O5B^iv^—Na7—O12	100.32 (5)
O21B—Mo3B—O4B	71.78 (4)	O20—Na7—O14^vii^	102.96 (6)
O8B—Mo3B—O4B	72.93 (3)	O13B^v^—Na7—O14^vii^	162.97 (5)
O17B—Mo4B—O12B	103.73 (6)	O5B^iv^—Na7—O14^vii^	83.91 (4)
O17B—Mo4B—O21B	101.14 (5)	O12—Na7—O14^vii^	87.50 (5)
O12B—Mo4B—O21B	101.57 (5)	O20—Na7—O1	80.53 (6)
O17B—Mo4B—O22B	99.57 (5)	O13B^v^—Na7—O1	84.83 (5)
O12B—Mo4B—O22B	98.32 (5)	O5B^iv^—Na7—O1	92.68 (5)
O21B—Mo4B—O22B	146.83 (4)	O12—Na7—O1	163.38 (6)
O17B—Mo4B—O4B	166.92 (5)	O14^vii^—Na7—O1	83.60 (5)
O12B—Mo4B—O4B	89.17 (5)	H51—O5—H52	105.0
O21B—Mo4B—O4B	73.69 (4)	H71—O7—H72	105.0
O22B—Mo4B—O4B	80.36 (4)	H81—O8—H82	104.9
O17B—Mo4B—O7B	85.57 (5)	H91—O9—H92	105.0
O12B—Mo4B—O7B	168.90 (5)	H101—O10—H102	105.0
O21B—Mo4B—O7B	82.23 (4)	H111—O11—H112	105.0
O22B—Mo4B—O7B	73.83 (4)	H121—O12—H122	105.0
O4B—Mo4B—O7B	81.85 (3)	H131—O13—H132	105.0
O13B—Mo5B—O18B	105.26 (6)	H141—O14—H142	105.0
O13B—Mo5B—O22B	96.53 (5)	H151—O15—H152	105.0
O18B—Mo5B—O22B	98.97 (5)	H161—O16—H162	105.0
O13B—Mo5B—O23B	99.76 (5)	H171—O17—H172	105.0
O18B—Mo5B—O23B	96.50 (5)	H181—O18—H182	104.9
O22B—Mo5B—O23B	153.69 (4)	H191—O19—H192	104.9
O13B—Mo5B—O3B	96.85 (5)	H201—O20—H202	104.9
O18B—Mo5B—O3B	157.26 (5)	H211—O21—H212	104.9
O22B—Mo5B—O3B	83.47 (4)	H11N—N1—H12N	109.5
O23B—Mo5B—O3B	74.21 (4)	H11N—N1—H13N	109.5
O13B—Mo5B—O7B	166.16 (5)	H12N—N1—H13N	109.5
O18B—Mo5B—O7B	85.72 (4)	H11N—N1—H14N	109.5
O22B—Mo5B—O7B	73.07 (4)	H12N—N1—H14N	109.5
O23B—Mo5B—O7B	87.07 (4)	H13N—N1—H14N	109.5
O3B—Mo5B—O7B	73.29 (3)	H21N—N2—H22N	109.5
O1B—P1B—O2B	112.22 (6)	H21N—N2—H23N	109.5
O1B—P1B—O4B	109.35 (6)	H22N—N2—H23N	109.5
O2B—P1B—O4B	108.15 (6)	H21N—N2—H24N	109.5
O1B—P1B—O3B	111.48 (6)	H22N—N2—H24N	109.5
O2B—P1B—O3B	106.11 (5)	H23N—N2—H24N	109.5
O4B—P1B—O3B	109.40 (5)	H31N—N3—H32N	109.4
O5B—P2B—O6B	111.49 (6)	H31N—N3—H33N	109.5
O5B—P2B—O7B	110.43 (6)	H32N—N3—H33N	109.5
O6B—P2B—O7B	109.17 (5)	H31N—N3—H34N	109.5
O5B—P2B—O8B	110.02 (6)	H32N—N3—H34N	109.5
O6B—P2B—O8B	106.37 (5)	H33N—N3—H34N	109.5
O7B—P2B—O8B	109.26 (5)	H41N—N4—H42N	109.5
O6^vii^—Na1—O5	151.31 (6)	H41N—N4—H43N	109.5
O6^vii^—Na1—O8^viii^	80.81 (5)	H42N—N4—H43N	109.5
O5—Na1—O8^viii^	85.76 (5)	H41N—N4—H44N	109.5
O6^vii^—Na1—O10B^vi^	81.44 (5)	H42N—N4—H44N	109.4
O5—Na1—O10B^vi^	78.23 (5)	H43N—N4—H44N	109.4
O8^viii^—Na1—O10B^vi^	106.45 (5)	H51N—N5—H52N	109.5
O6^vii^—Na1—O18	117.14 (6)	H51N—N5—H53N	109.5
O5—Na1—O18	90.43 (6)	H52N—N5—H53N	109.4
O8^viii^—Na1—O18	102.53 (6)	H51N—N5—H54N	109.5
O10B^vi^—Na1—O18	147.83 (6)	H52N—N5—H54N	109.5
O6^vii^—Na1—O15A^vii^	81.16 (4)	H53N—N5—H54N	109.5
O5—Na1—O15A^vii^	114.30 (5)		

Symmetry codes: (i) *x*, *y*+1, *z*; (ii) *x*+1, *y*, *z*; (iii) *x*, *y*, *z*+1; (iv) −*x*, −*y*+1, −*z*+1; (v) −*x*+1, −*y*+1, −*z*+1; (vi) −*x*, −*y*+2, −*z*+1; (vii) *x*−1, *y*, *z*; (viii) −*x*+1, −*y*+2, −*z*; (ix) *x*, *y*, *z*−1; (x) *x*, *y*−1, *z*.

### Hydrogen-bond geometry (Å, °)

**Table:**

*D*—H···*A*	*D*—H	H···*A*	*D*···*A*	*D*—H···*A*
O1—H11···O12B^ix^	0.86	2.02	2.8415 (18)	160
N1—H11N···O12A	0.87	2.07	2.750 (2)	135
O1—H12···O16A^vii^	0.86	2.55	3.383 (2)	164
N1—H12N···O20	0.87	2.13	2.963 (3)	161
N1—H13N···O18B^v^	0.87	2.37	2.909 (2)	121
N1—H14N···O23B^v^	0.87	2.29	2.922 (2)	130
O2—H21···O21B^xi^	0.86	2.29	3.1414 (17)	172
N2—H21N···O5A^vii^	0.87	2.10	2.8892 (19)	151
N2—H21N···O13A	0.87	2.59	3.146 (2)	123
O2—H22···O20A	0.86	2.07	2.9134 (18)	165
N2—H22N···O9A	0.87	2.20	3.036 (2)	162
N2—H23N···O1A	0.87	1.98	2.837 (2)	171
N2—H24N···O15A^vii^	0.87	2.06	2.9121 (19)	167
O3—H31···O23A^v^	0.86	1.86	2.7120 (16)	174
N3—H31N···O5B^iv^	0.87	1.95	2.8066 (19)	170
O3—H32···O14B^v^	0.86	2.00	2.8440 (16)	165
N3—H32N···O9B^v^	0.87	2.09	2.9533 (19)	173
N3—H33N···O1B^v^	0.87	2.04	2.8410 (19)	152
N3—H34N···O15B^iv^	0.87	2.19	3.017 (2)	159
O4—H41···O10^vii^	0.86	1.89	2.743 (2)	172
N4—H41N···O1A	0.87	2.00	2.8544 (19)	171
O4—H42···O23A	0.86	2.57	3.403 (2)	164
N4—H42N···O5A^vii^	0.87	2.02	2.8757 (19)	169
N4—H43N···O12A^v^	0.87	2.44	3.123 (2)	136
N4—H43N···O22A	0.87	2.26	2.9312 (19)	134
N4—H44N···O17A^v^	0.87	2.23	2.958 (2)	142
O5—H51···O9A	0.86	2.40	3.0367 (18)	132
O5—H51···O15B^vi^	0.86	2.42	3.201 (2)	151
N5—H51N···O1B^v^	0.87	2.03	2.8649 (19)	162
O5—H52···O2A	0.86	1.97	2.8118 (18)	167
N5—H52N···O5B^iv^	0.87	1.96	2.8251 (17)	175
N5—H53N···O13B^v^	0.87	2.55	2.9140 (18)	106
N5—H54N···O21B^iv^	0.87	2.45	3.0098 (18)	123
N5—H54N···O17B^ix^	0.87	2.28	3.0203 (19)	144
O6—H61···O10A	0.86	2.01	2.8234 (17)	159
O6—H62···O20B^xi^	0.86	1.84	2.6971 (16)	176
O7—H71···O3^v^	0.86	1.89	2.7347 (19)	167
O7—H72···O6A^xii^	0.86	2.09	2.9430 (16)	173
O8—H81···O6	0.86	1.87	2.7221 (19)	174
O8—H82···O2B^xii^	0.86	2.01	2.8642 (16)	171
O9—H91···O5A^xii^	0.86	1.90	2.7476 (18)	171
O9—H92···O13A^vi^	0.86	2.34	2.9118 (19)	124
O9—H92···O14B^vi^	0.86	2.58	3.0630 (18)	117
O1—H01···O9B^xii^	0.86	2.20	2.985 (2)	152
O10—H102···O6A	0.86	2.10	2.893 (2)	153
O11—H111···O17A^xiii^	0.86	2.58	2.987 (2)	110
O11—H112···O17A^xiii^	0.86	2.58	2.987 (2)	110
O11—H112···O21A	0.86	2.39	3.126 (2)	143
O12—H121···O9^x^	0.86	2.04	2.891 (2)	171
O12—H122···O18A^v^	0.86	2.01	2.834 (2)	160
O13—H131···O19A^x^	0.86	2.13	2.944 (2)	158
O13—H132···O2B^v^	0.86	2.12	2.904 (2)	151
O14—H141···O11	0.86	1.92	2.772 (2)	171
O14—H142···O6B^v^	0.86	2.17	2.9073 (18)	143
O15—H151···O6B^vi^	0.86	2.01	2.8578 (17)	169
O15—H152···O15B^vi^	0.86	2.37	3.0264 (18)	134
O15—H152···O9A	0.86	2.43	3.192 (2)	148
O16—H161···O13^xiv^	0.86	1.92	2.769 (2)	171
O16—H162···O20B^xi^	0.86	2.39	3.220 (2)	163
O17—H171···O1B^ix^	0.86	2.44	3.167 (2)	143
O17—H172···O16B^xi^	0.86	2.18	2.949 (2)	149
O18—H181···O1A	0.86	2.35	3.105 (2)	147
O18—H182···O11B^ix^	0.86	2.34	2.874 (2)	121
O19—H191···O21	0.86	2.48	3.270 (4)	154
O19—H192···O2	0.86	1.99	2.830 (3)	166
O20—H201···O21	0.86	1.87	2.716 (3)	166
O20—H202···O17A^v^	0.86	1.93	2.757 (2)	162
O21—H211···O1A	0.86	1.93	2.784 (3)	169
O21—H212···O18B^v^	0.86	2.44	2.992 (3)	123

Symmetry codes: (ix) *x*, *y*, *z*−1; (vii) *x*−1, *y*, *z*; (v) −*x*+1, −*y*+1, −*z*+1; (xi) *x*+1, *y*, *z*−1; (iv) −*x*, −*y*+1, −*z*+1; (vi) −*x*, −*y*+2, −*z*+1; (xii) −*x*+1, −*y*+2, −*z*+1; (xiii) −*x*+2, −*y*+1, −*z*+1; (x) *x*, *y*−1, *z*; (xiv) −*x*+1, −*y*+1, −*z*.
